# A Pilot Quantitative Study of Topographic Correlation between Reticular Pseudodrusen and the Choroidal Vasculature Using En Face Optical Coherence Tomography

**DOI:** 10.1371/journal.pone.0092841

**Published:** 2014-03-21

**Authors:** Dilraj S. Grewal, Jonathan Chou, Stuart D. Rollins, Amani A. Fawzi

**Affiliations:** Department of Ophthalmology, Northwestern University Feinberg School of Medicine, Chicago, Illinois, United States of America; University of Iowa, United States of America

## Abstract

**Purpose:**

To analyze the topographic correlation between reticular pseudodrusen (RPD) visualized on infrared reflectance (IR) and choroidal vasculature using en-face volumetric spectral-domain optical coherence tomography (SD-OCT).

**Methods:**

A masked observer marked individual RPD on IR images using ImageJ (NIH, Bethesda, MD). Using the macular volume scan (Cirrus, Carl Zeiss Meditec Inc, Dublin, CA), the RPE slab function was used to generate a C-scan of the most superficial choroidal vasculature. An independent masked grader created a topographic binary map of the choroidal vasculature by thresholding the en-face image, which was overlaid onto the IR map of RPD. For each IR image, ImageJ was used to generate a random set of dots as “control lesions”.

**Results:**

17 eyes of 11 patients (78±13.7 years) with RPD were analyzed. The average number of RPD lesions identified on IR images was 414±71.5, of which 49.6±4.3% were located overlying the choroidal vasculature, compared to 45.4±4.0% in controls (p = 0.014). 50.4±4.3% of lesions overlay the choroidal stroma, of which 76.5±3.1% were ≤3 pixels from the choroidal vessels. The percentage of RPD lesions located within ≤3 pixels from the choroidal vasculature was significantly greater than the percentage located ≥7 pixels away. (p<0.0001). Compared to controls (71.6±3.8%), RPD were more likely to be located ≤3 pixels away from choroidal vessels (p = 0.014). In contrast, control lesions were more likely to be ≥7 pixels away from choroidal vessels than RPD (9.1±1.9% vs. 4.8±1.2%, respectively, p = 0.002).

**Conclusions:**

Our analysis shows that RPD lesions follow the underlying choroidal vasculature. Approximately half the RPD directly overlay the choroidal vessels and the majority of the remaining lesions were ≤3 pixels (≤30microns) from the vessel edge, supporting the hypothesis that RPD maybe related to pathologic changes at the choroidal level.

## Introduction

Reticular pseudodrusen (RPD) were first described in 1990 as ‘pseudodrusen visible in blue light’ [1]. With the application of multimodal imaging and a higher sensitivity for imaging RPD [2]–[8], _ENREF_3_ENREF_8clinicians have gained an improved understanding of this entity and uncovered a higher prevalence of these lesions than previously thought. Cross-sectional studies have revealed that eyes with RPD are more likely to develop advanced lesions of age related macular degeneration (AMD), while longitudinal studies have shown a higher incidence of progression to late stage AMD among subjects with RPD compared to those without [2], [9]–[14]._ENREF_13

Despite its wide prevalence and high risk for progression, the exact location and pathogenesis of RPD lesions remain the subject of great dispute. In the original histopathologic report of an eye with clinically confirmed RPD [10], the choroid showed loss of medium sized vessels with constriction of the remaining choriocapillaris, along with increased stromal fibrosis, raising the possibility that choroidal changes may be associated with RPD. The choroid has recently become accessible to in vivo studies through application of spectral-domain optical coherence tomography (SD-OCT) and en face sections of the choroid [15]. We have recently explored this approach for quantitative choroidal vascular mapping [16]. This approach reconstructs the OCT data, which is traditionally viewed in cross sections (B-scans), and instead views it in a coronal direction (C-scans) with the capability to align the plane of these en face C-scans to either the plane of the internal limiting membrane or the RPE. Using this approach, our group quantified choroidal vascular density *in vivo*, with excellent correlation to previous histopathologic results [16], [17].

Furthermore, using this approach we found that all eyes with RPD show a lack of choriocapillaris, along with an increased density of stroma in the most superficial choroidal layers, which concurs with the evidence found in the original histopathologic report of RPD [10]. Sohrab et. al. performed a qualitative study of RPD lesions seen on infrared reflectance (IR) and en face SD-OCT which showed they lie on top of intervascular choroidal stroma, and that the hyper-reflective subretinal drusenoid deposits (SDD) seen on SD-OCT lie immediately adjacent to the reticular patterns on top of the adjacent choroidal vessels [18]. This finding was corroborated by Querques and colleagues who showed that RPD lesions on indocyanine green angiography (ICG) closely abutted the large choroidal vessels [19]. The choroidal theory, however, does not fully explain the growing body of evidence showing accumulation of subretinal drusenoid material in these eyes [4], [20]. One possible explanation that would explain the choroidal and subretinal manifestations of RPD is that choroidal fibrosis and ischemia could lead to the secondary derangement of the overlying retinal pigment epithelium (RPE) with impaired function leading to accumulation of photoreceptor outer segment and other debris above the RPE [18], [21].

In the current study, we wanted to utilize en face OCT of the choroidal vasculature to quantitatively and mathematically study the topographic correlation between RPD lesions on IR imaging and choroidal vessels, using automated image analysis and tools used in histopathologic studies of flat-mounted human donor eyes. Lengyel et. al. used this approach in histopathologic specimens to demonstrate that naturally autofluorescent drusen were located on top of the intercapillary pillars of the choriocapillaris [22]. Using a similar approach in the current study, we set out to quantitatively examine the hypothesis that RPD lesions follow the choroidal vasculature.

## Materials and Methods

The study was approved by the Institutional Review Board at Northwestern University, Feinberg School of Medicine and adhered to the tenets set forth by the Declaration of Helsinki. We retrospectively analyzed medical records of patients diagnosed with RPD at the Department of Ophthalmology, Northwestern Memorial Hospital between January 2012 and December 2012. The identification of RPD was based on the recognition of characteristic features on various imaging modalities as defined in previous reports [5]. Patients with a complete ophthalmological examination, including Infrared Reflectance (IR), fundus autofluorescence (FAF) imaging, and SD OCT (Cirrus high-definition OCT; HD-OCT, Carl Zeiss Meditec Inc, Dublin, CA USA) and the Spectralis HRA+OCT (Heidelberg Engineering Inc, Dossenheim, Germany), were included in this study.

RPD were defined by the peculiar yellowish reticular pattern at the macula, whose visibility was enhanced by IR reflectance appearing as groups of hyporeflective lesions interspersed against a background of mild hyperreflectance [18]. The criteria for the presence of RPD were applied to the entire portfolio of imaging studies for each patient. Previous studies have shown that IR is most sensitive for the detection of RPD compared to FAF and red-free images, and have used IR to show the presence of RPD crossing the central macula [5], [18].

Eyes with severe epiretinal membrane and macular scars, which precluded the identification and classification of RPD, were excluded. Only patients with high quality IR images that made RPD lesions easily identifiable were included. Since our analysis was based on percentages, eyes with fewer than 50 RPD lesions were excluded. The OCT scan with the best image quality or the most obvious display of the characteristic of interest was selected for each patient in the study.

### Image acquisition

Images were acquired on the Cirrus HD-OCT 4000 device (Carl Zeiss Meditec, Dublin, CA), using a super-luminescent diode at 840 nm, which achieves a 5 μm axial and a 15 μm transverse tissue resolution. The device captures 27,000 A-scans per second at 2 mm of depth. The images were viewed with the Cirrus HD-OCT software (version 6.0.1.24 Carl Zeiss Meditec Inc.). Imaging was performed using either a 200×200 or a 512×128 macular cube volume scan, the latter consisting of 128 equally spaced horizontal B-scans (each composed of 512 A-scans) over a 6-mm square grid. In the 512×128 scan protocol, the pixel separation in the horizontal direction is 6000/512 or 11.7 μm, while the pixel separation in the vertical direction is 6000/128 or 46.9 μm. The actual beam width or transverse resolution is ∼15 μm. The 200×200 macular cube volume scan consists of 200 equally spaced horizontal B-scans, also over a 6-mm square grid. This scan protocol allows 1024 pixels per A-scan (each pixel is 2000/1024 mm in depth), 200 A-scans per Fast B-scan and 200 B-scans per volume scan, providing a separation between A-scans of 30 μm. The pixel spacing in the axial direction for both cubes is 2000/1024 or approximately 2 μm, while the axial resolution is approximately 5 μm. The line scanning laser ophthalmoscope (SLO) feature obtained a registered IR fundus image for each data cube. Only volume scans free of motion artifact were selected for this study.

### En Face OCT Choroidal and Outer Retinal Sections

OCT volume scans were reviewed on Cirrus version 6.0 software using the advanced visualization feature. As previously described [18], [23], _ENREF_16the RPE feature was used to obtain en face slices, or C-scans, which were contoured based on each patient's RPE curvature. The horizontal section (slab thickness) was adjusted to ensure that neither the RPE band nor sclera were included in the C-scan slabs. En face choroidal slabs, 20 μm in thickness, were generated at the level of the most superficial choroidal vasculature and reviewed for each patient. By using the RPE slice overlay feature, these slabs were reviewed and registered to the Cirrus IR fundus image with guidance from the SD-OCT scan. In two patients in which SDD were clearly visualized on OCT sections, 10 μm thick en face slabs, were generated above the level of the RPE and just below the level of the external limiting membrane (ELM) to highlight the SDD which were seen as white circular spots. The choroidal slabs were thicker in order to provide enough structural information of the superficial choroidal vasculature. In contrast, the scans used to visualize the SDD were thinner in order to isolate the narrow space between the RPE and the ELM where SDD have been reported to be located. These images were subsequently compared to the IR RPD map to evaluate for a correlation between SDD and RPD distribution.

### Identification of RPD on IR Images

**Figure 1:**
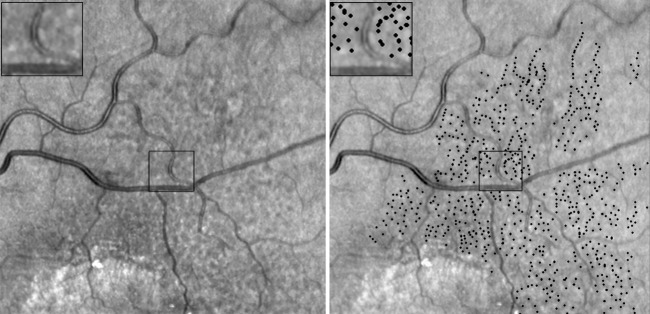
Infrared reflectance (IR) image of lesions: IR image (left), Reticular pseudodrusen (RPD) lesions highlighted by a masked retinal specialist using ImageJ software (NIH, Bethesda, MD) with inset of the area providing a magnified view (1a, right).

ImageJ software (NIH, Bethesda, MD) was used to perform the subsequent analysis [24]. A single investigator (AAF) used the paintbrush tool to individually mark each RPD lesion visualized on the IR reflectance image, using the characteristics described above. This investigator was masked to the en face OCT of the choroidal vasculature (Figure 1). Accuracy of lesion identification was studied using 5 randomly selected patients, where another masked reader (DSG) independently identified the RPD lesions.

Another independent grader performed the subsequent steps of the analysis (JC). The marked IR image was thresholded until only the RPD lesion markings were visible. The “find maxima” option (output type: single point) was used to identify all the lesions. The resulting map contained single pixel dots with a thresholded value of 255, each representing the center of an individual RPD lesion. The pixel values were divided by 255 so that each RPD was represented by a single pixel with a value of 1.

### Identification of Choroidal Vessels on en face C-Scans

**Figure 2:**
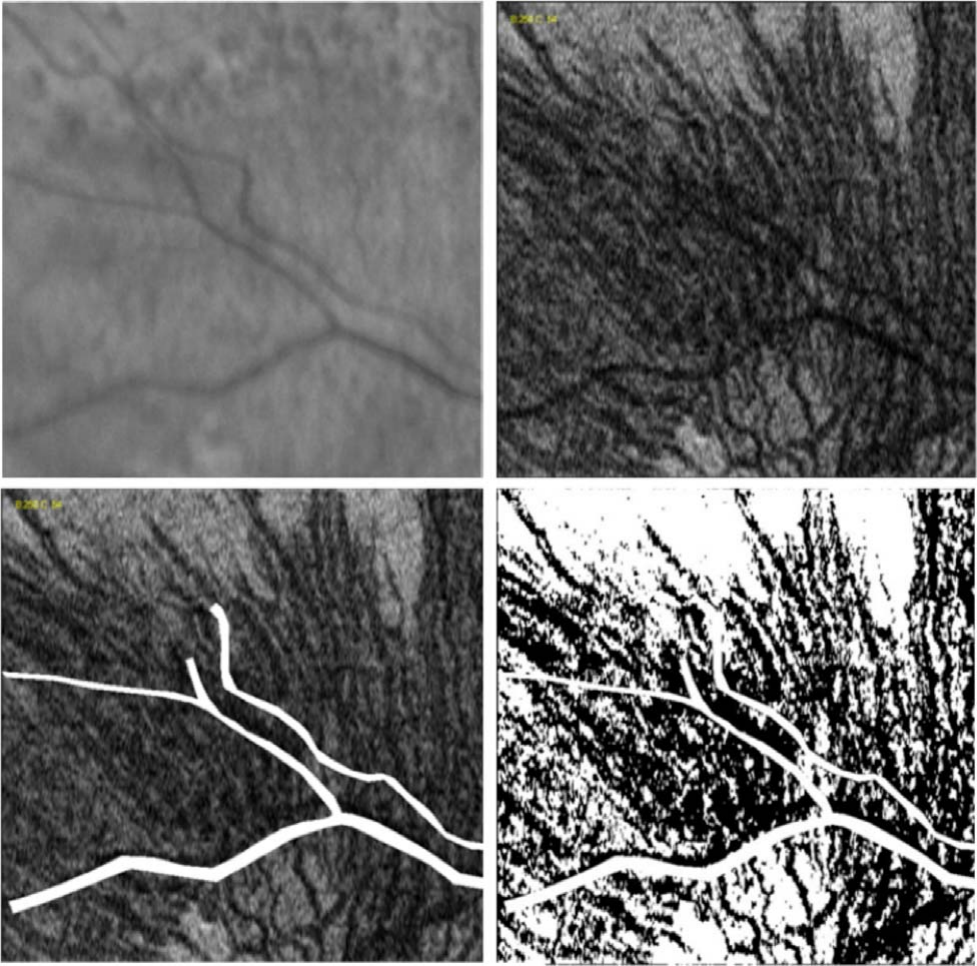
Infrared reflectance (IR) image (top left), En Face SD OCT Choroidal Sub-Layer C-Scan (top right). C-scan image with areas corresponding to the retinal vessels removed using ImageJ software (visible in white, bottom left), and pixel intensity thresholded C-scan using ImageJ software (choroidal vessels in black; bottom right)

The IR and C-scan images were manually overlaid and compared. Areas corresponding to retinal vessels on the C-scan image were removed (Figure 2, bottom left). All marked RPD lesions that corresponded to these removed areas were excluded from the analysis.

A topographic binary map of the choroidal vasculature was created by thresholding the en face C scan. Based on a previous study [16], a threshold value of 60 was found to be most effective at choroidal vascular selection, providing the best discrimination between vessels and stroma and this was applied to all the images in our study (Figure 2, bottom right). The resulting thresholded map had a binary set of pixels, which carried a value of either 255 (vessels, black) or zero (stroma, white).

### Computer-generated RPD control lesions

In order to study the possibility that the topographic correlation between RPD lesions and the choroidal vasculature occurs by chance, we used computer-generated “control RPD lesions”. For each combination of IR and C-scan, a set of random dots was generated using the “noise” function on ImageJ software and labeled as “control RPD lesion map” for subsequent comparisons. The total number of random set of dots equaled the total number of RPD lesions on the corresponding image so that an accurate comparison could be performed.

### Image Analysis

The percentage of RPD lesions located either over the choroidal vasculature or stroma was determined. The “image calculator” function was used to multiply the lesion map (both control and actual RPD lesions, each lesion carrying a pixel value of 1) and the thresholded choroidal C-scan images (pixel values of 255 or zero, representing either vasculature or stroma, respectively). The “histogram” function was used to define the total number of pixels at 255, representing the number of lesions that overlay the choroidal vasculature. The percentage of RPD lesions located either overlying the choroidal vasculature or the stroma was then compared to the corresponding percentage of random dots (control RPD lesion map). Further analysis was performed on the RPD lesions overlying the choroidal stroma in order to evaluate the proximity (in pixels) of these RPD lesions to the surrounding choroidal vasculature as compared to the control RPD lesions. The proximity of the RPD lesions was categorized into three groups; 1–3 pixels, 4–6 pixels, and ≥7 pixels away from the choroidal vasculature. The C-scan image was inverted so that vessels were white. The “distance map” function was applied and used for linear distance calculations. The “histogram” function was used to determine the percentage of RPD and random set of dots at each of the three distance groups.

### Statistical analysis

Statistical analysis was performed using Excel (Microsoft Excel 2007, Microsoft, Redmond, WA). A one-tailed student t-test with a p-value <0.05 was considered significant. Demographic data is presented as mean ± standard deviation, while the RPD analysis is presented as the mean ± standard error of the mean.

## Results

**Figure 3:**
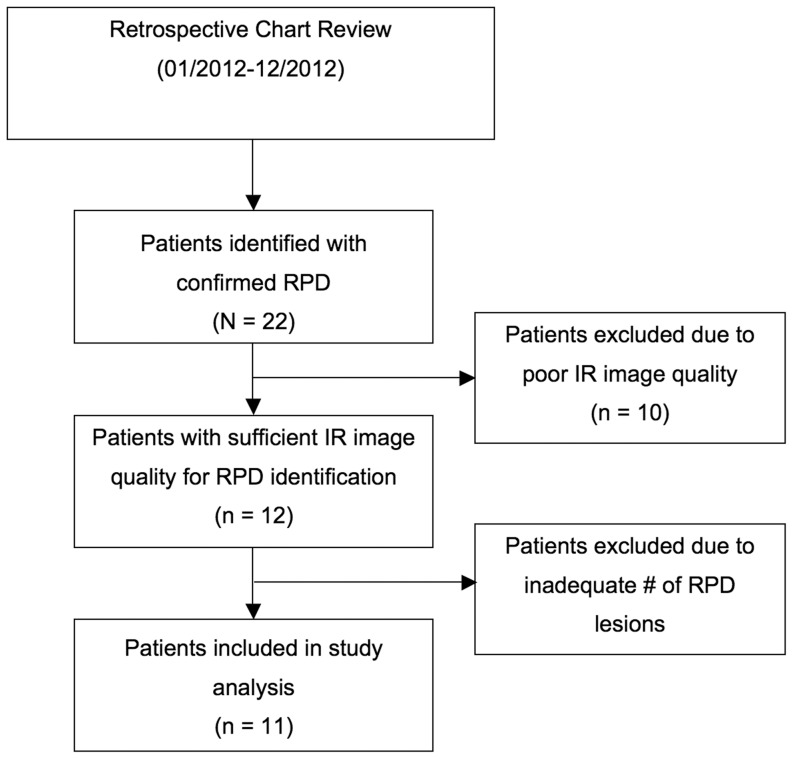
Flowchart demonstrating the review and selection process for the patients included in the study.

Retrospective review identified a total of 22 patients with RPD confirmed based on their entire imaging portfolio. Of these patients, 12 patients had adequate quality SD-OCT images and at least one IR image of sufficient quality to identify RPD lesions. Of these 12 patients, 1 was excluded due to an inadequate number of RPD lesions seen on IR reflectance (n<50). Figure 3 provides a flowchart of the patient selection process. A total of 17 eyes of 11 patients were included in the study. Of the 11 patients, 9 (81.8%) were female. The mean age was 78±13.7 years. The average number of lesions in each eye as identified on IR images was 414±71.5. For identification of RPD lesions on IR, there was good inter-rater reliability. The average intra-class correlation coefficient (ICC) was 0.98, p = 0.001 (95% CI; 0.812–0.998)

### The percentage of RPD lesions overlying the choroidal vasculature was significant compared to chance

**Figure 4:**
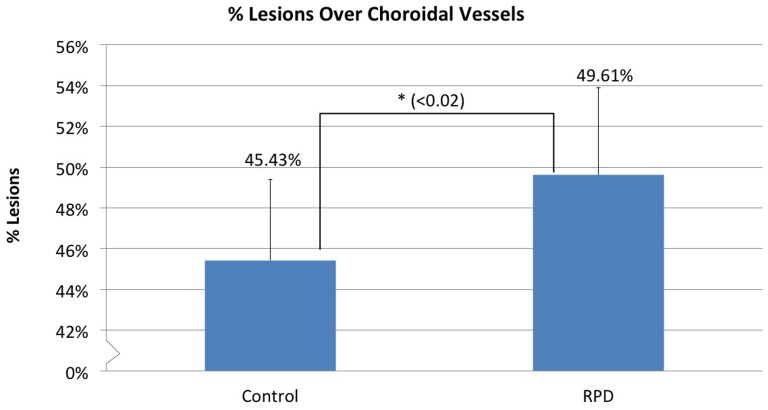
Percentage of RPD and Control RPD lesions (randomly placed dots) **overlying the choroidal vasculature.** An average of 49.6±4.3% of RPD lesions were overlying the choroidal vasculature which was significantly higher than the 45.4±3.9% of control lesions. (p = 0.014).

49.6±4.3% of RPD lesions were located directly over the choroidal vasculature compared to 45.4±3.9% of the random set of dots. This difference was 4.2±1.74%, which was statistically significant (p = 0.014), suggesting that a greater number of RPD are present overlying the choroidal blood vessels than could be mathematically explained by chance alone (Figure 4).

### RPD lesions located overlying the choroidal stroma were significantly in close proximity to the choroidal vasculature compared to chance

We categorized the proximity of the remaining RPD lesions to the choroidal vasculature measured in pixels into three groups (1–3 pixels, 4–6 pixels, and ≥7 pixels away from the choroidal vasculature).

**Figure 5:**
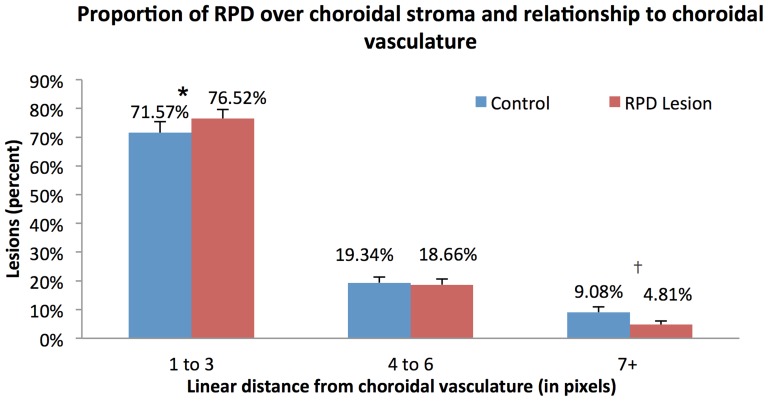
Distribution of the RPD and control RPD lesions (randomly placed dots) at different distances from the choroidal vasculature (measured in pixels). Among RPD and control lesions, there was a significantly greater percentage of RPD lesions lying between 1–3 pixels from the choroidal vessels (*, difference 4.2%, p = 0.014) while there was a significantly greater percentage of controls at ≥7 pixels (†, difference 3.6%, p = 0.002) away from the choroidal vessels.

Overall, 50.4±4.3% of RPD lesions overlay the choroidal stroma, of which 76.5±3.1% were located within 3 pixels away from the choroidal vessels, while 18.7±2.0% were located 4–6 pixels away and 4.8±1.2% were ≥7 pixels away from the choroidal vasculature. In contrast, among the random set of dots, 71.6±3.8% were within 3 pixels, 19.3±2.0% were within 4–6 pixels and 9.1±1.9% were located ≥7 pixels away from the choroidal vasculature. The percentage of RPD lesions located within 1–3 pixels from the choroidal vasculature was significantly greater than the percentage located ≥7 pixels away (p<0.0001). Comparing RPD to the random set of dots, we found a significantly greater percentage of RPD lesions located within 3 pixels (p = 0.014) of the choroidal vasculature and a significantly smaller percentage of RPD lesions located ≥7 pixels away (p = 0.002). There was a similar distribution of the RPD and control for the 4–6 pixels distance from the choroidal vasculature (p = 0.25) (Figure 5). These results suggest that RPD lesions overlying the choroidal stroma were more likely to be located within close proximity (1–3 pixels) of the choroidal vessels than could be explained by random chance.

### Lack of Topographic Correlation between RPD on IR and SDD on OCT

**Figure 6:**
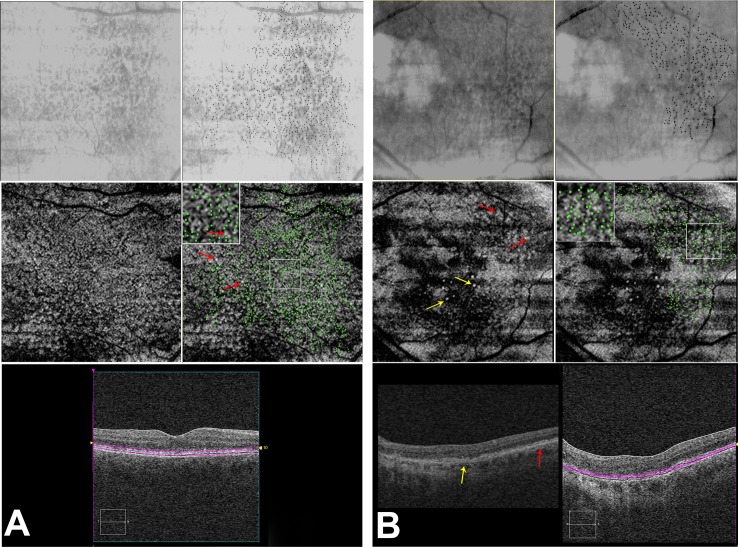
Correlation of RPD lesions on IR reflectance to the sub-retinal drusenoid deposits (SDD) on OCT and comparison of regular drusen with SDD on OCT. 6A: IR reflectance (top left), IR reflectance with individual RPD lesions marked in black (top right). En face OCT C-scan (middle left) taken at the level of photoreceptors, as shown on b-scan (bottom) showing the sub-retinal drusenoid deposits (SDD; white lesions, yellow arrows). Middle right panel shows an overlay of the RPD lesions from IR (green dots) onto the en-face scan demonstrating the relative lack of overlap between the SDD and the RPD, with the inset providing a magnified view. OCT B-scan (bottom right) highlighting in purple, the location of the en face section. 6B: shows a different eye, which has SDD as well as small hard drusen. Hard drusen are marked by yellow arrows on the C scan (middle left) and the B-scan (bottom left). On en face OCT, hard drusen appear as well defined distinct white lesions surrounded by a ring of hypo-reflectivity (yellow arrows on B-scan and en face images). In contrast, SDD (marked by red arrows on the B-scan and en face images) have relatively indistinct borders, occur in clusters and appear to blend into an isointense background. OCT B-scan (bottom right) highlighting. in purple, the location of the en face section.

In an effort to explore the correlation between SDD and RPD in the patients in this study, we analyzed two eyes in which the SDD were most visible on en face images. RPD were highlighted on the IR image (Figure 6 A and B, top right, black dots) and these were overlaid onto the en face map of SDD, obtained at the level of the photoreceptors (Figure 6 A and B, middle right, green dots). A relative lack of correlation was seen between RPD visualized on IR and the SDD (Figure 6B, middle row, red arrows). En face slabs for SDD were created at the level of the discrete collections of hyperreflective material above the RPE, in the subretinal space (bottom).

### Macular Cube Parameters and Distance Calibration

When we export either a 200×200 or a 512×128 into the “Cirrus Advanced Visualization” application, the software automatically resizes the image for display so that the transverse anatomical relationships are the same for 512×128 as for 200×200. Hence, c-scan images obtained in our study would give similar pixel measurements in ImageJ. Each exported c-scan had 600×600 pixels, in the x-y directions, respectively. Given this information, for a 6 mm×6 mm cube original dimension, each pixel in our measurements correlates to 10 microns, whether in 200×200 or 512×128 cube.

**Figure 7:**
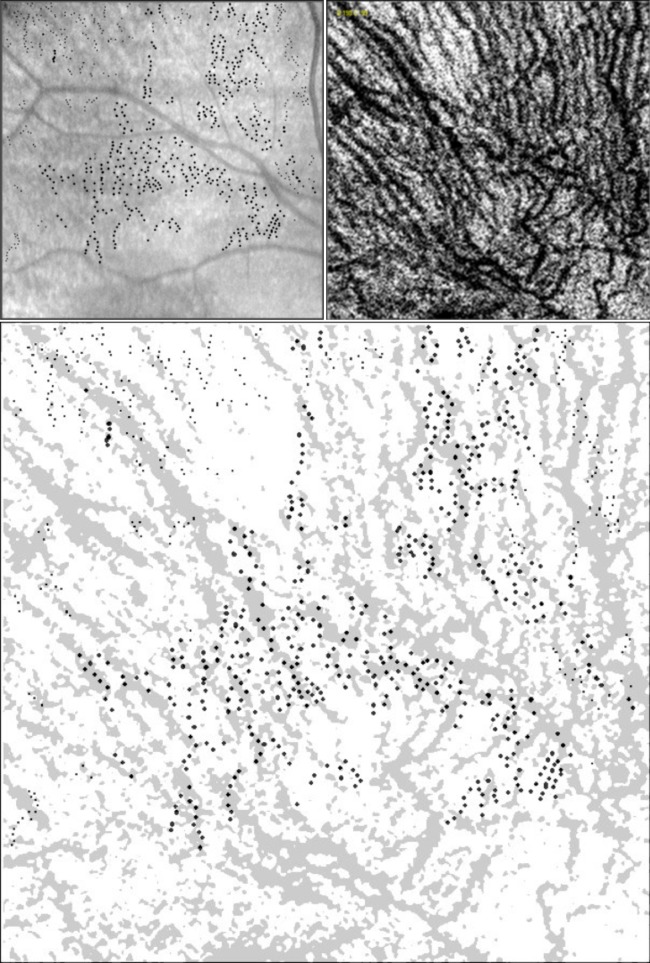
RPD lesions correlate with choroidal vasculature. IR reflectance RPD lesions shown as black dots (top left) and en face OCT C-scan (top right). Overlay of the RPD lesions and the thresholded C-scan highlighting the choroidal vasculature show an association between the location of the RPD lesions and the choroidal blood vessels (bottom). Of the 751 RPD lesions in this image, 31% were over the choroidal vasculature (n = 233). Of the lesions over the stroma (n = 518), 57.34% were between 1–3 pixels, 28.76% between 4–6 pixels, and 13.9% were ≥7 pixels away from the choroidal vasculature.

Overall, our data suggests that the location of RPD lesions correlates with the choroidal vasculature based on the finding that the majority of the lesions are located either directly overlying or immediately adjacent to the choroidal vessels (within 1–3 pixels, correlating to 10–30 microns; Figure 7). In addition, at a distance of ≥7 pixels (≥70 microns) away from the choroidal vessels, we are more likely to find random dots (control RPD lesions) than RPD lesions.

## Discussion

This study was designed to quantitatively investigate the choroidal correlations of RPD lesions using en face OCT sections through the sub-RPE space overlaid onto IR reflectance fundus images. Using a high-density OCT map allowed us to create a map of the choroidal vasculature in the entire macula, providing a more consistent and precise correlation with IR images. By comparing the percentage of RPD lesions to control RPD lesions (software-generated random dots) located over the choroidal vasculature or stroma, we show that RPD lesions follow the path of the underlying choroidal vasculature. Overall, our results demonstrated that while RPD lesions were almost evenly distributed between the choroidal stroma and the vasculature, they closely followed the outline of the large choroidal vessels. Additionally, the majority of RPD lesions overlying the stroma (76.5±3.1%) were located within 3 pixels (<30 microns actual distance) of the choroidal vasculature (p = 0.014). Furthermore, the lack of correlation between RPD seen on IR and SDD on OCT further confirm that reticular pseudodrusen are more complex than previously thought, with pathologic changes occurring both above and below the RPE [25].

We set out to explore the choroidal RPD correlations *in vivo*, in an attempt to shed more light on the exact location and pathogenesis of the RPD lesions. The first histopathological study in a donor eye with clinical diagnosis of RPD postulated that choroidal vascular disease was involved in RPD. These authors based their theory on findings of significant loss of the small vessels of the middle choroidal layer and increased fibrosis in the choroid between the large choroidal veins [10]. Our group recently reported choroidal abnormalities in eyes with RPD using en face OCT in eyes with RPD in the form of loss of choriocapillaris signature, along with increased choroidal stroma in the most superficial choroidal slabs, suggesting choroidal involvement in RPD [16]. Further clinical support for potential choroidal ischemic pathology in RPD includes choriocapillaris filling defects in these eyes on ICG imaging [5], as well as the recent finding of predilection of RPD lesions to choroidal vascular watershed zones [26].

A comprehensive choroidal vascular theory for RPD pathogenesis would need to explain the presence of SDD seen on SD-OCT, which appear as round or triangular hyperreflective deposits localized between the RPE and photoreceptors and occasionally extending into the external limiting membrane or the outer plexiform layer [6], [19], [21]. These deposits do not seem to correspond to the IR lesions, but are found adjacent to lesions, as shown on IR registered to SD-OCT [18] and ICG registered to SD-OCT and IR imaging [19], a finding which we further confirmed using registered subretinal OCT slabs in this study (Figure 6 A and B). A visual inspection revealed that the majority of the RPD on IR did not overlap with the SDD on the en face image. However our overlay method is limited since we were not able to visualize earlier stages of SDD (limited to outer segments) [4], which may have led us to underestimate the earlier stages of SDD.

Curcio et al. recently proposed a biologic explanation for the predilection of these subretinal drusenoid deposits to the perifovea, a region of high rod density, related to perturbation of lipoprotein transport mechanisms between rod outer segment and the RPE [25]. While intriguing, this theory does not account for the choroidal changes seen on various imaging modalities and in histopathologic studies. Furthermore, unlike the IR hyporeflective lesions, which are a hallmark appearance for RPD, SDD may be more prevalent than previously thought, and may not be specific to eyes with clinical evidence of RPD. This was recently shown by Sarks et al. who showed that histopathologic evidence of subretinal drusenoid material not only occurs in eyes with clinical RPD, but more interestingly, they demonstrated these deposits in eyes with AMD but without a clinical diagnosis of RPD [8].

An understanding of the pathogenesis of RPD would be facilitated if their clinical correlates or genetic risk factors were distinct from other forms of AMD. However, clinically, RPD lesions seen on IR may frequently coexist in eyes with various lesions of AMD. In a cross-sectional study of Japanese patients, the prevalence of RPD was 83% in eyes with retinal angiomatous proliferation, 50% in geographic atrophy, 9% in typical AMD, and 2% in polypoidal choroidal vasculopathy [27]. Similar to the clinical overlap between RPD and various AMD disease phenotypes, the genetic risk factors also appear to overlap, though this remains controversial. In a small cross sectional study of patients of European American ancestry, Smith et. al. [11] found an enhanced risk for RPD in association with at-risk SNP rs10490924 of ARMS2 (Odds Ratio 1.73). In contrast, homozygosity for Y402H strongly associated with the absence of RPD (11.9% of RPD patients compared to 37.5% without RPD, p<0.001). This finding was corroborated in cross sectional study of a Japanese population by Ueda-Arakawa [27] who found a higher frequency of the at-risk SNP rs10490924 in ARMS2 in patients with RPD, (p = 0.007). On the other hand, in the largest study to date, Puche et. al. compared genetic risk alleles between 105 patients with RPD, 414 AMD patients without RPD and 430 healthy controls and found that AMD patients with RPD shared the same major genetic factors as AMD without RPD. [28] While the presence of genetic risk disparities between RPD and AMD may require larger population studies to elucidate, it is interesting to note that a variety of molecules associated with AMD risk are highly concentrated in the choroid. Components of the alternate and classical complement pathways, including complement factor H, are highly concentrated in the choroid with significantly lower levels in the RPE and neural retina [29], [30]. ARMS2, whose function remains unknown, shows robust immune labeling in the inter-choriocapillaris pillars [31], putative “entrapment sites” of drusen accumulation [22], [32]. Choroidal atrophy and fibrosis have been hypothesized to lead to derangement of the RPE and secondary accumulation of photoreceptor OS above the RPE in RPD [19]. Photoreceptor involvement is further supported by a recent study demonstrating that eyes with RPD had significantly reduced macular sensitivity measured using microperimetry as compared to eyes with typical drusen [33].

The tendency for drusen to accumulate over choroidal stroma or inter-capillary pillars has intrigued researchers for the last several decades [22], [34]. The most widely accepted theory for this tendency relates to impeded clearance of cellular debris in areas without choroidal vascular lumen [35]. Other theories include the unidentified differential adhesion characteristics in the intercapillary region, and the possibility that dendritic cells may play a role, since they can be recruited between blood vessels and act as a focus for debris originating in the RPE [36]. Based on immunohistochemical and histopathological studies, Mullins et. al. [37] _ENREF_29suggested that some drusen constituents (e.g., plasma proteins such as amyloid P component and prothrombin) may leak out of the choroidal vessels and into the extracellular space adjacent to the RPE, where they bind to one or more ligands associated with developing drusen. More recently, these authors showed a negative correlation between drusen volume and choriocapillaris density, further suggesting choroidal involvement in early AMD [38]. Lengyel et. al. showed that small drusen tend to accumulate overlying the intercapillary pillars [22]. Similar to Lengyel, our findings suggest a predilection for RPD to be located overlying or closely adjacent to the choroidal vessels. Compared to a random set of dots, the difference, although small, was statistically significant. This conclusion is further supported when comparing the percentage of RPD lesions located within 1–3 pixels from the choroidal vasculature with the percentage located ≥7 pixels away (p<0.0001) where the difference was more prominent. One thing to note is that the smaller choroidal vessels, choriocapillaris and residual “ghost” or non-perfused choriocapillaris, which are likely smaller than a horizontal pixel (11 μm), were under-estimated by our approach[16]. This study uses our prior work as a framework to provide a quantitative analysis of RPD and choroidal vasculature correlation [16]. Unique to our investigation were the masked and automated comparison of the RPD on IR reflectance images to the choroidal C-scans on OCT using a predetermined threshold applied to all images and the use of a random set of dots for each IR image as “control RPD lesions”.

Our study has some limitations related to the lateral resolution of currently available SD-OCT imaging devices (∼15 μm). The average size of the choriocapillaris on histopathology is ∼20 μm, [39] whereas the choriocapillaris diameter measured using OCT in control eyes has been shown to be on average ∼50 microns [16]. This limitation makes current commercial OCT systems inadequate for accurate choriocapillaris quantification. Recent research has shown that phase-variance OCT is a promising imaging method that can yield higher choroidal resolution and possibly allow differentiation between arterioles and venules, as well show perfused versus non-perfused choriocapillaris [40]. Furthermore, although we attempted to target the choriocapillaris layer by identifying the section immediately below the RPE, it is possible that the most superficial C-scan in RPD includes larger choroidal vessels (e.g. Sattler's and Haller's layer) interspersed with the atrophic choriocapillaris [16]. We compared thinner C-scans as well (2,4,6,8 and 10 μm in thickness) to try to limit inclusion of the larger choroidal vessels, but the image quality was not adequate for the current analysis. Higher resolution imaging technologies like adaptive optics SLO might have sufficient lateral resolution to delineate the choriocapillaris, though they maybe limited by their inability to image deeper than the RPE. Future studies using swept source OCT that provides higher-penetration and enhanced imaging of the choroidal vasculature with improved choroidal segmentation may improve our sensitivity to detect RPD [41]. The small sample size is another limitation of this study, which was dictated by the strict criteria we applied for RPD diagnosis, the minimum number of RPD lesions and requisite IR and SD-OCT image quality. Additionally, we had no examples of histopathology to correlate to the changes noted on SD-OCT. The use of arbitrary pixel intensity cut-offs for distinguishing between vessel and stroma is another limitation, but this approach has been used previously by our group with excellent correlation to histopathology and is similar to the software analysis employed in quantifying choroidal vasculature in histopathologic flatmount analysis [17].

Based on our finding, we are able to conclude that RPD lesions tend to follow the path of the choroidal vasculature. We could not determine whether RPD lesions seen on IR exclusively lie on top of the choroidal stroma. In order to determine this, one would need to only include the smallest RPD lesions. In the current study, we did not outline the entire RPD lesion; rather, we used the center of the lesion, marked by a 2-pixel spots. Hence, it is possible that larger RPD lesions partly overlay the vessels as well as the choroidal stroma, or alternatively that RPD lesions that fell over the vessels were merely abutting them rather than overlying their center.

In conclusion, using en face SD-OCT and registered IR images, we quantified and confirmed the close relationship between RPD lesions and the larger choroidal vessels, supporting choroidal vascular involvement in the pathogenesis of these lesions. En face OCT of the choroid is emerging as a powerful tool to study the choroidal vasculature *in vivo*, allowing us to perform quantitative and detailed analyses, similar to histopathologic studies.[15], [16], [18] With its wider availability and non-invasive nature, this approach will have exciting implications for the ability to perform longitudinal studies to further our understanding of choroidal vascular involvement in different variants of AMD.
